# Generation of clonal zebrafish line by androgenesis without egg irradiation

**DOI:** 10.1038/srep13346

**Published:** 2015-08-20

**Authors:** Jilun Hou, Takafumi Fujimoto, Taiju Saito, Etsuro Yamaha, Katsutoshi Arai

**Affiliations:** 1 Faculty and Graduate School of Fisheries Sciences, Hokkaido University, 3-1-1 Minato, Hakodate, Hokkaido 041-8611, Japan; 2 Nanae Fresh-Water Laboratory, Field Science Center for Northern Biosphere, Hokkaido University, 2-9-1 Sakura, Nanae, Kameda, Hokkaido 041-1105, Japan

## Abstract

Generation of clonal zebrafish will facilitate large-scale genetic screening and help us to overcome other biological and biotechnological challenges due to their isogenecity. However, protocols for the development of clonal lines have not been optimized. Here, we sought to develop a novel method for generation of clonal zebrafish by androgenesis induced by cold shock. Androgenetic zebrafish doubled haploids (DHs) were induced by cold shock of just-fertilized eggs, and the eggs were then heat shocked to double the chromosome set. The yield rate of putative DHs relative to the total number of eggs used was 1.10% ± 0.19%. Microsatellite genotyping of the putative DHs using 30 loci that covered all 25 linkage groups detected no heterozygous loci, confirming the homozygosity of the DHs. Thus, a clonal line was established from sperm of a DH through a second cycle of cold-shock androgenesis and heat-shock chromosome doubling, followed by genetic verification of the isogenic rate confirming the presence of identical DNA fingerprints by using amplified fragment length polymorphism markers. In addition, our data provided important insights into the cytological mechanisms of cold-shock–induced androgenesis.

Zebrafish (*Danio rerio*), a small tropical teleost, have been widely used as a model organism in various fields of scientific research for many years^1^,^2^,^3^. Clonal lines are important for obtaining stable, reproducible results in biological studies. To establish clonal lines in fish, the first step is to obtain the first generation of doubled haploids (DHs), which are completely homozygous gynogenetic or androgenetic diploids generated by inhibition of the first cleavage event to produce a double chromosome set after parthenogenetic development. The second step is production of gynogenetic or androgenetic diploids from isogenic gametes generated by the DH. Compared with inbred strains, which require at least 20 generations of sib mating, clonal lines generally only require about two generations for establishment. Streisinger *et al*. first reported the development of clonal lines in zebrafish by inducing gynogenesis followed by inhibition of the second polar body release of eggs obtained from gynogenetic DH females^4^. Additionally, Mizgirev and Revskoy generated clonal zebrafish lines by two successive production cycles of gynogenetic diploids^5^. Within the clonal line, they successfully transplanted tumor cells between adult fish without severe immune reactions^5^.

Androgenetic DHs inherit chromosomes only from the male parent and are completely homozygous. Therefore, they are useful for rapidly establishing clonal lines^6^,^7^,^8^, mapping quantitative trait loci^9^, investigating the effects of mitochondria or cytoplasm on development and growth^10^, and screening and identifying genes carrying recessive mutations^11^. In recent next-generation sequencing analysis, DHs are helpful for improvement in *de novo* assembly of teleost genomes^12^. As a first step to inducing androgenetic DHs, the eggs are genetically enucleated using irradiation with gamma rays, X-rays, or UV rays before fertilization with intact sperm. In zebrafish, androgenesis can be achieved by irradiation of eggs with high-energy X-rays^13^ or short-wave UV rays^11^ to inactivate their nuclei genetically. However, the irradiation method requires special facilities or equipment to ensure safety, as well as protective solutions, such as artificial ovarian fluid to prevent the eggs from drying out and losing fertilization ability during irradiation. However, a recent study described the use of cold-shock androgenesis without irradiation in a different teleost fish, the loach *Misgurnus anguillicaudatus*^14^.

In this study, we report the successful induction of androgenetic DHs and establishment of clonal line without any irradiation of eggs in zebrafish by using the cold-shock method described for the loach.

## Results

### Optimization of the temperature of cold-shock treatment

**Figure 1:**
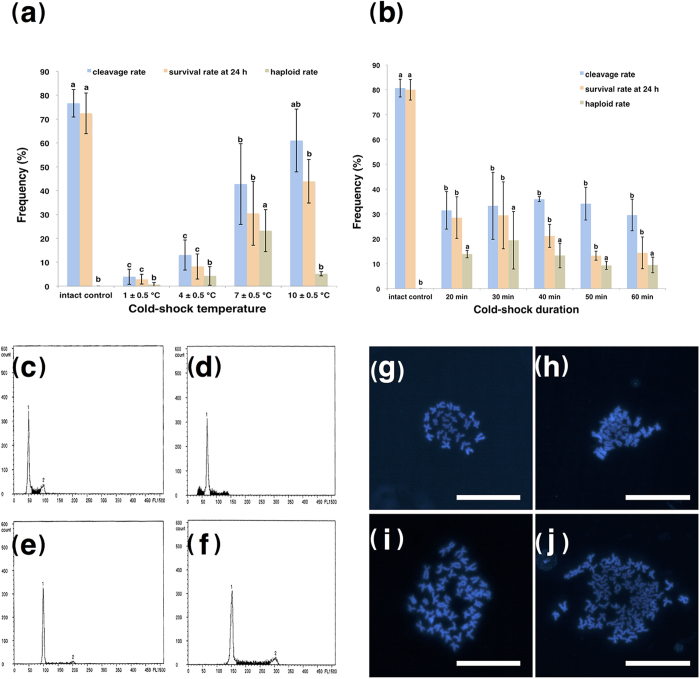
Induced haploid androgenesis by cold-shock in zebrafish (*Danio rerio*). (**a**) Cleavage rate, survival rate at 24 h, and haploid rate of just-fertilized eggs cold-shocked at 1 ± 0.5 °C, 4 ± 0.5 °C, 7 ± 0.5 °C, and 10 ± 0.5 °C for 30 min. Different letters above the columns denote significant differences as determined by one-way ANOVA and Duncan’s multiple comparisons (*P* *<* 0.05). (**b**) Cleavage rate, survival rate at 24 h, and haploid rate of just-fertilized eggs cold-shocked at 7 ± 0.5 °C for 20, 30, 40, 50, or 60 min. Different letters above the columns denote significant differences as determined by one-way ANOVA and Duncan’s multiple comparisons (*P* < 0.05). (**c–j**) Relative DNA content and metaphase chromosomes of different ploidy larvae hatched from intact control eggs and eggs subjected to cold shock at 7 ± 0.5 °C for 30 min. (**c**) 1N abnormal larvae from the cold-shock group; (**d**) 1.3N abnormal larvae from the cold-shock group; (**e**) 2N normal larvae from the intact control group; (**f**) 3N abnormal larvae from the cold-shock group; (**g**) 1N abnormal larvae with 25 chromosomes from the cold-shock group; (**h**) 1.3N abnormal larvae with 32 chromosomes from the cold-shock group; (**i**) 2N normal larvae with 50 chromosomes from the intact control group; (**j**) 3N abnormal larvae with 75 chromosomes from the cold-shock group. Scale bars denote 10 μm.

In order to optimize the cold-shock temperature, just-fertilized eggs (within 10 s after fertilization [AF]) were exposed to different temperatures (1, 4, 7, or 10 °C) for 30 min. Eggs that were subjected to cold shock at 1, 4, or 7 °C showed significantly lower cleavage rates when compared with the intact control (76.61% ± 5.77%, *P* < 0.05; Fig. 1a). In contrast, the cleavage rate of eggs exposed to cold shock at 10 °C (61.03% ± 13.15%) was not significantly different from that of the intact control (*P* > 0.05; Fig. 1a). Intact control eggs had the highest survival rate at 24 h AF (72.38% ± 8.52%), and this survival rate was significantly higher than those of eggs in all cold-shocked groups (*P* < 0.05; Fig. 1a). Among the cold-shocked groups, survival rates of eggs exposed to 7 °C (30.46% ± 13.39%) and 10 °C (43.92% ± 9.10%) at 24 h AF were significantly higher than those of eggs exposed to 1 °C (2.87% ± 2.05%) and 4 °C (8.23% ± 5.28%; Fig. 1a).

Both normal and abnormal larvae were observed in all groups except the 4 °C group, which had only abnormal larvae (Table S1). Flow cytometric analysis indicated that all larvae in the intact control group were diploid. In contrast, all normal larvae from the 1 °C and 7 °C groups were triploid. In eggs shocked at 10 °C, in addition to triploid larvae, six diploid and one hyperdiploid larvae were observed among the normal larvae. Haploid larvae only appeared in some of the abnormal larvae from all cold-shock groups, together with other ploidies, such as diploid, triploid, and hypo- or hyperdiploid (Table S1).

Eggs in the 7 °C cold-shock group had the highest haploid rate (23.23% ± 8.81%; *P* < 0.05; Fig. 1a). However, haploid rates among other cold-shock groups were not significantly different (*P* > 0.05; Fig. 1a). The results indicated that 7 °C was the optimum cold-shock temperature to induce androgenetic haploidy in zebrafish.

### Optimization of the duration of cold-shock treatment

Next, we optimized the duration of cold-shock treatment (7 °C) of just-fertilized eggs, examining durations of 20, 30, 40, 50, and 60 min. Intact control eggs had significantly higher cleavage (80.66% ± 3.57%) and survival (79.99% ± 4.15%) rates at 24 h AF when compared with eggs exposed to cold-shock treatment (*P* < 0.05; Fig. 1b). The cleavage rates (29.60% ± 6.33% to 35.97% ± 1.00%) and survival rates (13.24% ± 1.82% to 29.46% ± 13.45%) decreased abruptly at 24 h AF in the cold-shock groups, but the differences among these groups were not significant (*P* > 0.05). Additionally, no significant differences were observed in haploid rates among cold-shock groups (Fig. 1b).

Both normal and abnormal larvae were observed after all durations of cold shock, and only normal larvae were observed in the intact control group (Table S2). Flow cytometry indicated that all normal larvae in the intact control group were diploid (Table S2 and Fig. 1e). In eggs exposed to cold shock for 30, 40, 50, or 60 min, all the normal larvae were triploid. Five of six normal larvae in the 20-min cold-shock group were triploid, and one was diploid. The majority of the abnormal larvae from different cold-shock duration groups were haploid (Fig. 1c); however, some were diploid, triploid (Fig. 1f), hypodiploid (Fig. 1d), or hyperdiploid (Table S2). The results of chromosome spreads showed that the diploid larvae from the intact control group had a chromosome number of 2N = 50 (Fig. 1i), while the haploid, hypodiploid (e.g., 1.3N), and triploid larvae from the cold-shock groups had chromosome numbers of 1N = 25 (Fig. 1g), 1.3N = 32 (Fig. 1h), and 3N = 75 (Fig. 1j), respectively. These were consistent with the results of flow cytometry.

### Determination of the paternity of haploid androgenetic eggs

Microsatellite genotyping was carried out at four loci: *Z7576*, *Z6010*, *Z9708*, and *Z11786*^15^ to verify the all-paternal inheritance of haploid individuals. In the diploid progeny hatched after cold-shock treatment, one maternally derived allele and one paternally derived allele were detected at each locus, while two maternally derived alleles and one paternally derived allele were detected in the triploid progeny. In contrast, only the paternally derived allele was detected at each locus in haploid progeny (Table S3). Therefore, the haploid progeny hatched after cold-shock treatment was found to be androgenetic. The results of ploidy status and microsatellite analysis indicated that haploid androgenesis was successfully induced without any irradiation, using only cold-shock treatment of eggs in zebrafish. The haploid yield rate achieved here was similar to that reported by Corley-Smith *et al*.^13^, suggesting that cold-shock treatment of just-fertilized eggs is as effective as X-ray irradiation in the induction of haploid androgenesis in zebrafish.

### Cytological mechanism of cold-shock androgenesis

To elucidate the cytological mechanism of cold-shock androgenesis in zebrafish, we observed the early developmental process of cold-shocked eggs using histological sections. The developmental process and morphological features of normal fertilized zebrafish eggs are similar to those of other teleosts (Fig. S1)^16^,^17^. Eggs were fertilized at the metaphase of the second meiosis, and the second polar body was then released. Female and male pronuclei were formed and fused to generate the zygote. Eventually, the eggs began the first mitosis.

**Figure 2:**
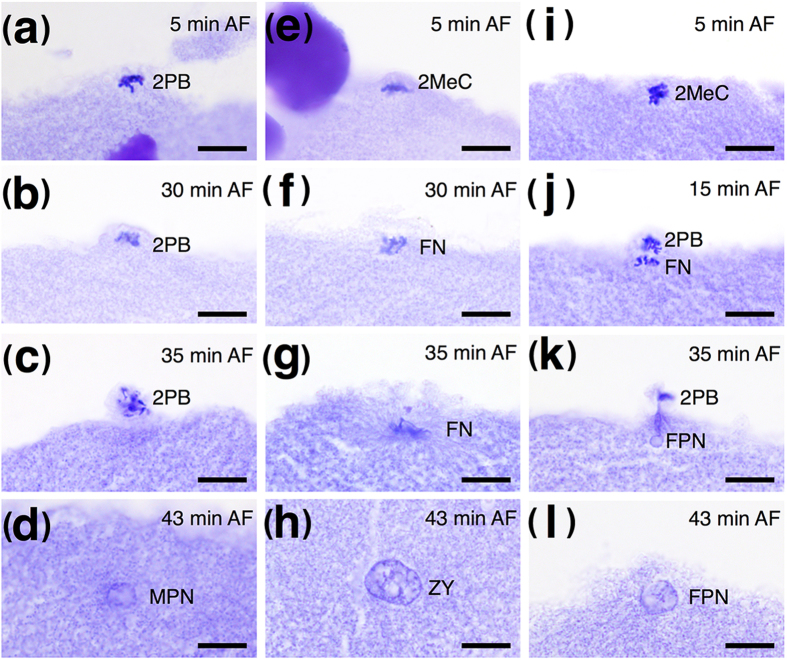
Histological observation of zebrafish (*Danio rerio*) androgenetic-induced eggs from 5 to 43 min after fertilization (AF). (**a–d**) androgenetic haploid type; (**e–h**) triploid type; and (**i–l**) diploid or aneuploid type. (**a**) 5 min AF, the spindle was destroyed by cold-shock treatment, and part of the blastodisc swelled up with all the chromosomes; (**b**) 30 min AF, the blastodisc swelled further with all the chromosomes; (**c**) 35 min AF, the swelled blastodisc released as second polar body with all chromosomes; (**d**) 43 min AF, only one pronucleus was observed in the egg; (**e**) 5 min AF, part of the blastodisc swelled; (**f**) 30 min AF, the blastodisc swelled further with no chromosomes; (**g**) 35 min AF, few parts of the blastodisc were released, and all chromosomes remained in the egg; (h) 43 min AF, the zygote was formed by the fusing of female and male pronuclei; (**i**) 5 min AF, the unswelled blastodisc; (**j**) 15 min AF, parts of the blastodisc swelled up with some chromosomes, and other chromosomes were found at unswelled parts of the blastodisc; (**k**) 35 min AF, the second polar body was released with some chromosomes, and the unreleased chromosomes in the egg developed into the female pronucleus; (**l**) 43 min AF, the female pronucleus became bigger. Scale bars denote 10 μm. Abbreviation: 2PB, 2nd polar body; 2MeC, 2nd meiotic chromosomes; FN, female nucleus; FPN, female pronucleus; MPN, male pronucleus; ZY, zygote.

Histological section of cold-shocked eggs allowed us to categorize nuclear and blastodisc behaviors into three types as follows. (1) The second meiotic chromosomes were enclosed and released together with second polar body. Only male nuclei existed in the egg. Eggs of this type initiated androgenesis, resulting in haploid progeny (Fig. 2a–d). (2) The second meiotic chromosomes were enclosed, but the second polar body was released without chromosomes. The unreleased diploid female nuclei and haploid male nuclei fused together, resulting in triploid progeny (Fig. 2e–h). (3) The second polar body was released with a certain number of chromosomes. The number of chromosomes released could be 25 (half of diploid zebrafish chromosomes), less than 25, or more than 25. If the released chromosome number were 25, the unreleased haploid female nucleus would fuse with the male nucleus, resulting in diploid progeny. If the released chromosome number were not 25, the egg would develop abnormally, resulting in aneuploid progeny (Fig. 2i–l). The results we obtained here are the same as those reported in the loach^14^, indicating that both zebrafish and loach share common mechanisms for cold–shock-induced androgenesis.

### Chromosome doubling of haploid androgenetic progeny via heat-shock treatment

**Figure 3:**
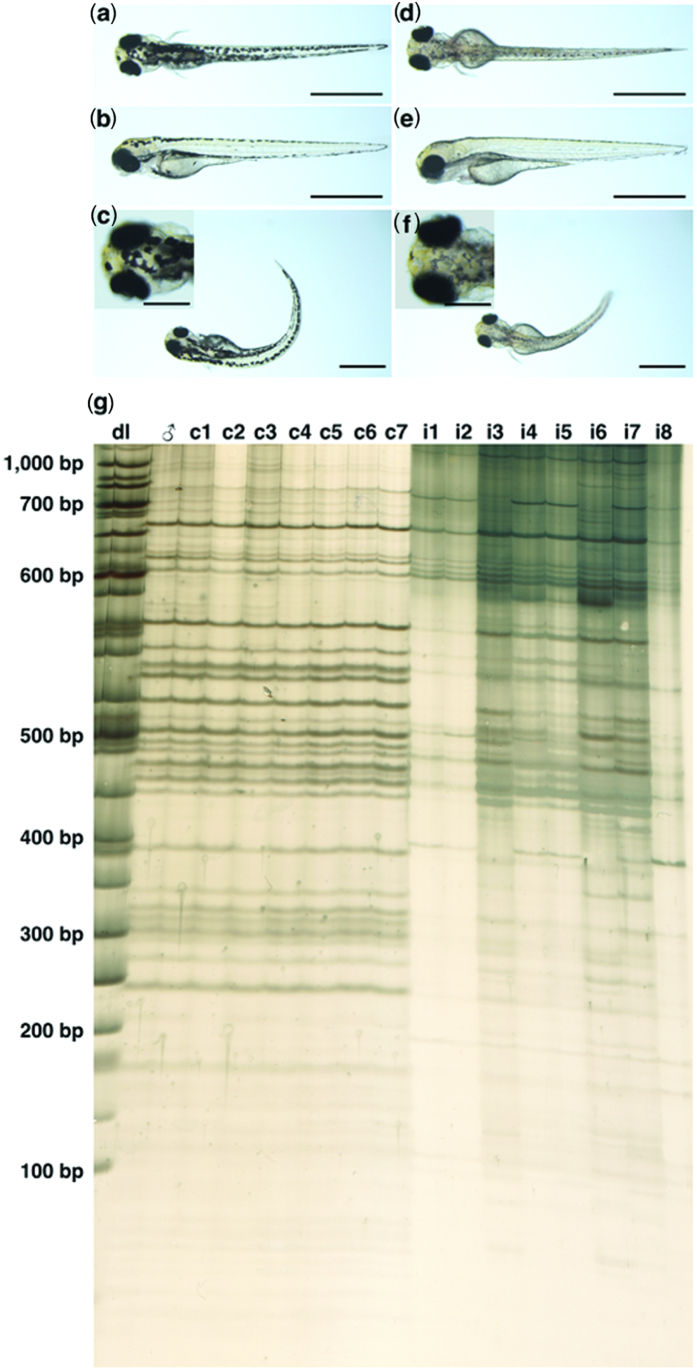
Induced androgenetic doubled haploids and clonal line in zebrafish (*Danio rerio*). (**a–f**) The four different types of larvae hatched from eggs subjected to cold-shock treatment for 30 min at 7 ± 0.5 °C, followed by heat-shock treatment at 43 min AF. (**a**) and (**b**) Normal wild-type larva; (**c**) abnormal wild-type larva; (**d**) and (**e**) normal golden-type larva; and (**f**) abnormal golden-type larva. Scale bar denotes 1 mm. (**g**) DNA fingerprints were determined by amplified fragment length polymorphism using the selective primer combination E-AGC/M-CTT. dl: DNA ladder; ♂: golden-type male doubled haploid whose sperm was used for androgenesis induction; c1–c7: normal golden-type diploid progeny hatched from androgenesis (clonal fish); i1–i4: female intact control; i5–i8: male intact control.

Next, we attempted to restore the diploidy of androgenetic haploid progeny by chromosome doubling using heat-shock treatment, as previously described by Corley-Smith *et al*.^13^. Males with the golden phenotype, which is recessive to the wild-type phenotype, were used as a visible marker of androgenesis to identify the all-male inheritance^18^. After hatching, four types of larvae appeared: (1) normal wild-type larvae (Fig. 3a,b); (2) abnormal wild-type larvae (Fig. 3c); (3) normal golden-type larvae (Fig. 3d,e); and (4) abnormal golden-type larvae (Fig. 3f). We designated the normal golden-type larvae as putative DHs that could survive and calculated the yield rate as the frequency of golden-type larvae relative to the total eggs used. A yield rate of 1.10% ± 0.19% was obtained from four batches of induction (Table 1).

The homozygosities of eight putative DHs were determined at 46–118 days old using 30 microsatellite loci from 25 linkage groups of zebrafish (two markers for linkage groups 1, 5, 6, 8, and 17, and one marker each for the remaining 20 linkage groups). For all the 30 loci tested, each golden-type fish was completely homozygous, and no heterozygous loci were detected (Table S4). Therefore, normal golden-type zebrafish hatched from cold- and then heat-shocked eggs were DH androgenetic progeny.

The sex of androgenetic DHs (n = 19) were examined by dissecting the fish after they died (n = 14) or collecting sperm by squeezing (n = 5). The examined individuals were all males; no females appeared. In contrast, no biased sex ratio was found in normal diploids reared in our facility.

### Establishment of a clonal line

To establish clonal line, we induced the second cycle of androgenesis using cold- and heat-shock with the sperm of the DH androgenetic male. In total, seven normal diploid progeny with the golden phenotype were obtained from this DH androgenetic male. These progeny, together with the male parent, were analyzed using DNA fingerprinting with AFLP to evaluate clonality. For all 64 primer sets used, they had identical DNA fingerprints, yielding a band sharing index (BSI) of 1. In contrast, in the intact control, the BSI of 31 primer sets was 0.76 ± 0.13 (Fig. 3g and Table S5). This result indicated that the seven progeny hatched from androgenesis were isogenic to the male parent and were therefore members of the clonal line.

## Discussion

In this study, we report, for the first time, the generation of androgenetic haploids and DHs in zebrafish using only thermal shock. The yield rate of androgenetic haploids using the optimum cold-shock temperature was 23.23% ± 8.81%. Corley-Smith *et al*. reported yield rates of androgenetic haploids ranging from 8% to 28% following induction by X-ray irradiation of eggs^13^. Ungar *et al*. reported successful induction of androgenetic haploids in zebrafish by irradiation of eggs using UV; however, they did not provide the yield rate^11^. The haploid yield rate we reported here was comparable to that reported by Corley-Smith *et al*.^13^. This finding suggests that cold-shock treatment of just-fertilized eggs is as effective to induce haploid androgenesis in zebrafish as X-ray irradiation. Moreover, cold-shock treatment does not require special facilities or solutions for irradiation and will therefore be more convenient for routine use. Additionally, the yield rate of normal androgenetic DHs was only 1.10% ± 0.19% in our present study, which was similar to that reported by Corley-Smith *et al*. (1.3%–2.1%)^13^.

An androgenetic clonal line was established by using two cycles of cold–shock-induced androgenesis, followed by heat-shock inhibition of the first mitosis. This is the first time that an androgenetic clonal line has been established in zebrafish. In teleosts, androgenetic clonal lines have been established only in common carp^19^, nile tilapia^20^, amago salmon^7^, and rainbow trout^21^,^22^. Relative to the total number of fish species, the percentage of species with successful generation of androgenetic clonal lines is quite low. However, this lack of androgenetic clonal lines can be overcome by the high reproductive performance of fish. An adult female zebrafish can spawn about 1000 eggs in 20–30 days, and sperm can be collected from male zebrafish every day. Therefore, for one batch of androgenetic induction, about 10 DHs can be obtained. The number of DHs will increase with multiple batches of induction using different females and males. After three months, the second cycle of androgenesis can be induced with the sperm of the DH, therefore allowing the establishment of clonal lines. With this method, only three months is needed for the establishment of clonal lines; this is much shorter than the full-sib mating method, which takes at least 60 months for the required 20 generations. Thus, androgenesis has obvious advantages over the full-sib mating method in terms of time required to establish the isogenic line.

In our study, we produced androgenetic DHs (n = 19) that were all males; no females appeared. Similar results, where all progeny were males, were reported in five androgenetic DHs by Corley-Smith *et al*.^13^. This lack of production of females suggests the involvement of a female heterogametic system (ZW female, ZZ male) in the sex determination mechanism in zebrafish. However, skewed sex ratios have been observed in gynogenetic DH zebrafish; for example, Streisinger *et al*. observed variable sex ratios in clones, such as predominantly females/males^4^, whereas Hörstgen-Schwark observed the exclusive occurrence of males in all gynogenetic experiments^23^. In developmental study, the number of primordial germ cells in the early zebrafish larvae has an important effect on the sexual differentiation in which a threshold number of primordial germ cells is required for stabilization of the ovarian fate^24^. But the genetic context of the sex determination mechanism in zebrafish has not been fully elucidated. To explain complicated results of sex ratio analysis, Liew and Orbán proposed a mechanism of polygenic sex determination, in which multiple sex-determining loci may be distributed throughout whole genome^25^. The recent studies using genetic analysis of restriction site associated markers proposed that chromosome 4 has functional and structural properties consistent with a sex chromosome, suggesting a ZW/ZZ sex determination mechanism in zebrafish^26^,^27^. However, the sex linked loci located on the tip of chromosome 4 was modified in the domesticated zebrafish strains^27^. In addition to the chromosome 4, three other separate genomic regions (chromosome 3, 5, 16) have also been identified as influencing sex determination, with variations between strains or even within families^26^,^28^. These results of the sex ratio using DHs and genetic study suggest that the sex of zebrafish is mainly determined by a ZZ/ZW system driven by the strong sex determination region on chromosome 4, but in the case of lacking it, other genomic regions have effects on sex determination, working as a mechanism of polygenic sex determination.

In conclusion, we report, for the first time, the induction of viable androgenetic DH progeny and an androgenetic clonal line in zebrafish without irradiation of eggs; our method used only a combination of cold- and heat-shock treatments. By eliminating the requirement for specialized protection media and irradiation, this method simplifies the process of androgenetic clonal line induction in zebrafish and has the potential to be widely used in other fish species.

## Materials and Methods

### Ethics

This study was performed according to the Guide for the Care and Use of Laboratory Animals in Hokkaido University. All animal experiments were approved by the animal study ethical committee of Hokkaido University (Approval number 19-2).

### Fish and gamete collection

Wild-type and golden-type zebrafish were reared in the Aquarium Room of the Environment Control Experiment Building in the Faculty of Fisheries Sciences, Hokkaido University. Zebrafish were acquired from the Nanae Freshwater Laboratory, Hokkaido University. The fish were maintained at 28.5 °C with a 14-h light/10-h dark photoperiod. On the evening before the experiment, male and female zebrafish were placed into separate mesh chambers in a 10-L aquarium at 28.5 °C. On the morning of the experiment, zebrafish were anesthetized with 0.1% 2-phenoxyethanol. The sperm was collected from each male by using 10-μL pipette tips and was placed in a 1.5-mL microtube containing 50 μL Hank’s solution (0.137 M NaCl, 5.4 mM KCl, 0.25 mM Na_2_HPO_4_, 0.44 mM KH_2_PO_4_, 1.3 mM CaCl_2_, 1.0 mM MgSO_4_, and 4.2 mM NaHCO_3_) on ice. Eggs were collected on polyvinylidene chloride film (Saran Wrap: Asahi Kasei Co. Ltd., Tokyo, Japan) stretched over the bottom of a 35-mm plastic dish. For each batch, at least three wild-type females and three wild-type or golden-type males were used.

### Optimization of the temperature and duration of cold-shock treatment

Eggs and sperm were mixed in a culture-dish that covered with polyvinylidene chloride film, and then add a small amount of ambient tap water (28.5 ± 0.5 °C) to activate the eggs and sperm (starting time counting at this moment). The fertilized eggs were transferred using a glass pipet into containers (the cone base of a 50-mL conical tube was cut and covered with mesh) floated in a Styrofoam box containing 6 L of cold tap water within 10 s AF. The eggs were subjected to cold-shock treatment for 30 min at temperatures of 1 ± 0.5, 4 ± 0.5, 7 ± 0.5, or 10 ± 0.5 °C. In our experiments, all temperatures were accurate within 0.5 °C.

To optimize the cold-shock duration, just-fertilized eggs were cold-shocked at 7 °C for 20, 30, 40, 50, or 60 min. After cold-shock treatment, the eggs were transferred within their containers to another Styrofoam box containing 6 L of ambient tap water (28.5 ± 0.5 °C) for 180 min AF. The eggs were then transferred to 90-mm plastic dishes (one group per plastic dish) and incubated at 28.5 °C until 24 h AF. For the intact control group, we used just-fertilized eggs that had been transferred to a container in a Styrofoam box containing 6 L of ambient tap water (28 ± 0.5 °C) and incubated until 150 min AF.

### Chromosome doubling of haploid androgenetic progeny via heat-shock treatment

Just-fertilized eggs were first subjected to cold-shock at 7 ± 0.5 °C for 30 min and then transferred to a water bath at 28.5 ± 0.5 °C for 13 min. The eggs were then heat shocked at 41.4 ± 0.5 °C for 2 min and returned to 28.5 ± 0.5 °C, according to the procedures described by Corley-Smith *et al*.^13^.

To calculate the induction rate of normal androgenetic DHs, eggs from wild-type females were first fertilized by sperm from golden-type males and then subjected to sequential cold and heat shock. Normal hatched larvae without melanophores were designated as putative DHs, and the induction rate was calculated as the proportion of normal golden larvae relative to the total number of eggs used.

### Cleavage, survival, and haploid rates

The cleavage rate was calculated as the proportion of cleaved eggs relative to the total number of eggs used at 4 h AF. The survival rate was calculated as the proportion of surviving embryos relative to the total number of eggs used at 24 h AF. The haploid rate was calculated as the proportion of haploid larvae relative to the total number of eggs used.

### Determination of ploidy levels and preparation of chromosomes

Somatogenesis-stage embryos around 24 h AF were further analyzed for determination of relative DNA content using flow cytometry (PA-II, Partec GmbH, Münster, Germany), according to the procedures described by Fujimoto *et al*.^29^. In the ploidy analysis, the embryos were categorized into two types by their embryonic development at 24 h AF: normal or abnormal. To prepare chromosome spreads, embryos (24 h AF) that had been cold shocked at 7 ± 0.5 °C for 30 min were manually dechorionated using jeweler’s forceps and then transferred into 10^−2^ M colchicine for 90 min at 28.5 °C. Embryos were then transferred into 4.26 × 10^−2^ M trisodium-citrate for 8 min at room temperature, and yolks were removed. Deyolked embryos were then transferred to ice for an additional 8 min. The embryos were fixed in Carnoy’s solution (methanol:acetic acid ratio, 3:1) and stored in a freezer overnight. Chromosome preparations were performed according to the procedures described by Westerfield^30^.

### Paternity and homozygosity

To determine paternity, eggs from one wild-type female were fertilized with sperm from one wild-type male. Within 10 s of fertilization, the eggs were cold-shocked at 7 ± 0.5 °C for 30 min to induce androgenesis. After cold-shock treatment, the embryos were incubated at 28.5 °C. At approximate 24 h AF, each embryo was first digested with 85 μL of solution A (CyStain DNA 2 step, Cod. 05-5005, Partec GmbH) for 15 min. Next, 15 μL of the digested solution was stained with 500 μL of solution B (CyStain DNA 2 step, Cod. 05-5005, Partec GmbH) and analyzed by flow cytometry. The remaining 70 μL of digested solution was used for DNA extraction.

Ten haploid, 10 diploid, and 10 triploid larvae were genetically analyzed, along with the female and male parents, using four zebrafish microsatellite DNA loci: *Z7576*, *Z6010*, *Z9708*, and *Z11786*^15^.

To determine the homozygosity of androgenetic DHs, eight golden-type zebrafish ranging from 46 to 118 days old, that had hatched from cold- and heat-shock treatment, were first analyzed using flow cytometry to confirm the presence of diploidy and then analyzed genetically using 30 microsatellite loci (covering 25 linkage groups).

DNA extraction and microsatellite genotyping were performed according to the methods described by Shimoda *et al*.^15^.

### Histological sections

For the intact control, eggs were artificially fertilized with sperm and then incubated in ambient water at 28.5 °C ambient water. The fertilized eggs were fixed from 5 to 45 min AF (time interval, 5 min) in Bouin’s solution. The unfertilized eggs were also fixed.

For the androgenesis group, eggs were fixed at 5, 15, and 30 min AF during the cold-shock treatment; after the cold-shock treatment, eggs were fixed at 35, 40, and 43 min AF.

After a 3-h fixation, eggs were washed and stored in 80% ethanol. Eggs were subsequently dehydrated in a graded series of ethanol and butanol, embedded in paraffin, sectioned serially to 6-μm thickness, and stained with hematoxylin-eosin. For each stage, at least 20 eggs were used.

### Clonal line induction and AFLP

Androgenesis was induced with sperm from one 4-month-old golden-type DH according to the above protocol. AFLP was performed according to a previously described method^31^. Sample DNA was digested with *Eco*RI and *Mse*I and ligated with an adaptor using T4 ligase. The ligation products were diluted 5- or 10-fold with double-distilled water (DDW) for pre-amplification polymerase chain reaction (PCR). After pre-amplification, the products were diluted 25-fold with DDW and then amplified with selective primers. The selective-amplification products were electrophoresed on 6% denaturing polyacrylamide gels and visualized by silver staining. For clonal line, seven progeny together with the male parent were analyzed using 64 primer sets; for the intact control, four males and four females from one outbred family were analyzed with 31 primer sets (Tables S5 and S6).

### Data analysis

Data for optimization of cold-shock temperature and duration and for chromosome doubling in haploid androgenotes are shown as means ± standard deviations (SDs), based on at least triplicate experiments. Data were analyzed with one-way analysis of variance (ANOVA) followed Duncan’s multiple comparisons (*P* *<* 0.05). The band sharing index (BSI) was calculated with AFLP data according to the following formula: BSI = 2N_ab_/(N_a_ + N_b_), where N_a_ and N_b_ are the band numbers in individuals a and b, and N_ab_ is the band number shared by a and b^32^. All statistical analyses and BSI calculations were performed in R software^33^.

## Additional Information

**How to cite this article**: Hou, J. *et al*. Generation of clonal zebrafish line by androgenesis without egg irradiation. *Sci. Rep*. **5**, 13346; doi: 10.1038/srep13346 (2015).

## Supplementary Material

Supplementary Information

## Figures and Tables

**Table 1:** Normal androgenetic doubled haploid induction rate (♀: wild-type, ♂: golden-type) in zebrafish (*Danio rerio*).

**Batch**	**Total egg no.**	**Number of cleavage embryos (frequency %)**	**Number of normal golden-type progeny (frequency %)**
1	778	216 (27.76)	9 (1.16)
2	824	202 (24.51)	7 (0.85)
3	781	301 (38.54)	8 (1.02)
4	725	197 (27.17)	10 (1.38)
Mean ± SD		229 ± 29(29.50 ± 5.36)	9 ± 1(1.10 ± 0.19)
