# Supplementary material for: Generation of clonal zebrafish line by androgenesis without egg irradiation
Source: Sci Rep. 2015 Aug 20;5:13346. doi: 10.1038/srep13346 (PMC4542340; doi:10.1038/srep13346)
Supplement: Supplementary Information [file srep13346-s1.pdf]

Supplemental Information

Title: Generation of clonal zebrafish line by androgenesis without egg irradiation

Jilun Hou[a], Takafumi Fujimoto[b], *, Taiju Saito[c], Etsuro Yamaha[d], Katsutoshi Arai[b]

Supplemental Figure

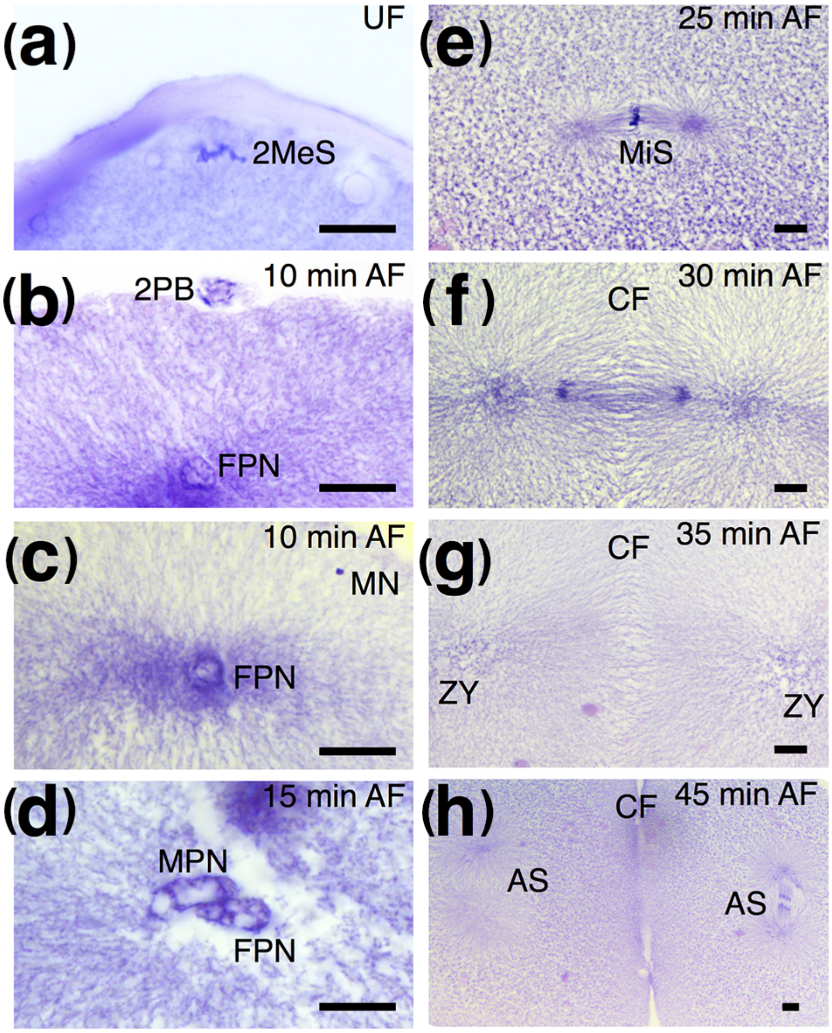

Figure S1. Histological sections of intact control eggs from zebrafish (*Danio rerio*). (a) Unfertilized eggs at metaphase of the second meiosis; (b) 10 min after fertilization (AF), second polar body was released, and the egg nucleus existed in the egg cytoplasm underneath the polar body; (c) 10 min AF, the condensed sperm nucleus and egg nucleus in the blastodisc; (d) 15 min AF, decondensed female and male pronuclei; (e) 25 min AF, metaphase of the first cleavage; (f) 30 min AF, anaphase of the first cleavage; (g) 35 min AF, prophase of the second cleavage; (h) 45 min AF, anaphase of the second cleavage. Scale bars denote 10 µm. Abbreviation: 2MeS, 2nd meiotic spindle; 2PB, 2nd polar body; FPN, female pronucleus; MN, male nucleus; MPN, male pronucleus; MiS, mitotic spindle; CF, cleavage furrow; ZY, zygote; AS, anaphase spindle.

Supplemental Tables

Table S1

External appearance and ploidy status of progeny from groups subjected to different temperatures during cold-shock treatment (30 min) in zebrafish (*Danio rerio*).

Treatment	External appearance	Number of larvae	Ploidy status				
			1N	2N	3N	Hypo-2N	Hyper-2N
Intact control	Normal	80	0	80	0	0	0
	Abnormal	16	0	16	0	0	0
1°C, cold-shock	Normal	2	0	0	2	0	0
	Abnormal	3	1	0	0	0	2[a]
4°C, cold-shock	Normal	0	0	0	0	0	0
	Abnormal	14	7	0	1	3[b]	3[c]
7°C, cold-shock	Normal	3	0	0	3	0	0
	Abnormal	47	39	0	1	6[d]	1[e]
10°C, cold-shock	Normal	30	0	6	23	0	1[f]
	Abnormal	44	8	8	17	7[g]	4[h]

[a] 2.3N and 2.8N
[b] 1.2N, 1.3N and 1.6N
[c] 2.4N, 2.5N and 3.4N
[d] 1.2N (3), 1.3N (2), and 1.5N
[e] 2.6N
[f] 2.6N
[g] 1.2N, 1.4N, 1.6N (3), and 1.7N (2)
[h] 2.2N (2), 2.6N, and 2.7N

Table S2

External appearance and ploidy status of progeny from groups subjected to different durations of cold-shock treatment (7°C) in zebrafish (*Danio rerio*).

Treatment	External appearance	Number of larvae	Ploidy status				
			1N	2N	3N	Hypo-2N	Hyper-2N
Intact control	Normal	104	0	104	0	0	0
	Abnormal	0	0	0	0	0	0
20 min cold-shock	Normal	6	0	1	5	0	0
	Abnormal	61	34	12	1	10[a]	4[b]
30 min cold-shock	Normal	7	0	0	7	0	0
	Abnormal	46	33	2	2	5[c]	4[d]
40 min cold-shock	Normal	6	0	0	6	0	0
	Abnormal	43	29	3	4	2[e]	5[f]
50 min cold-shock	Normal	4	0	0	4	0	0
	Abnormal	19	16	0	1	2[g]	0
60 min cold-shock	Normal	3	0	0	3	0	0
	Abnormal	21	16	0	2	0	3[h]

[a] 1.2N (4), 1.3N (2), 1.5N, 1.6N (2), and 1.7N
[b] 2.3N, 2.4N, 2.6N, and 2.8N
[c] 1.3N, 1.4N, 1.6N (2), and 1.8N
[d] 2.4N (3) and 6N
[e] 1.2N and 1.5N
[f] 2.3N, 2.6N (2), and 2.7N (2)
[g] 1.3N and 1.5N
[h] 2.2N and 2.4N (2)

Table S3

Microsatellite genotypes at four loci in haploid, diploid, and triploid progeny following cold-shock treatment (7°C, 30 min) in zebrafish (*Danio rerio*).

			Progeny from cold shock treatment		
Locus (LG)[a]	Female	Male	Haploid	Diploid	Triploid
Z7576	*208/246*	*121/121*	*121*: 10	*208/121*: 3	*208/246/121*: 10
(LG 12)				*247/121*: 7	
Z6010	*123/161*	*175/179*	*175*: 7	*123/175*: 3	*123/161/175*: 6
(LG 17)			*179*: 3	*123/179*: 3	*123/161/179*: 4
				161/175: 2	
				161/179: 2	
Z9708	*214/218*	*172/286*	*172*: 5	*214/172*: 1	*214/218/172*: 4
(LG 20)			*286*: 5	*218/172*: 1	*214/218/286*: 6
				214/286: 5	
				218/286: 3	
Z11786	*152/152*	*140/164*	*140*: 7	*152/140*: 5	*152/140*: 4
(LG 25)			*164*: 3	*152/164*: 5	*152/164*: 6

Abbreviation: LG, linkage group.
[a] See Shimoda *et al.* [15].

Table S4

Microsatellite genotyping of putative androgenetic doubled haploids (DHs) with the golden phenotype in zebrafish (*Danio rerio*).

Locus (LG)[a]	DH1	DH2	DH3	DH4	DH5	DH6	DH7	DH8
Z1781 (LG 1)	*200*	*200*	*200*	*200*	*200*	*200*	*200*	*200*
Z8874 (LG 1)	*150*	*150*	*150*	*150*	*150*	*150*	*150*	*150*
Z644 (LG 2)	*230*	*230*	*230*	*230*	*230*	*230*	*230*	*230*
Z9408 (LG 3)	*186*	*238*	*280*	*238*	*280*	*280*	*280*	*238*
Z7629 (LG 4)	*238*	*186*	*188*	*188*	*186*	*282*	*188*	*188*
Z896 (LG 5)	*228*	*205*	*205*	*228*	*228*	*228*	*205*	*228*
Z3314 (LG 5)	*126*	*126*	*126*	*126*	*126*	*126*	*126*	*126*
Z740 (LG 6)	*232*	*235*	*235*	*232*	*232*	*235*	*235*	*235*
Z1050 (LG 6)	*138*	*138*	*144*	*144*	*144*	*138*	*138*	*144*
Z8495 (LG 7)	*240*	*240*	*240*	*240*	*240*	*240*	*240*	*240*
Z1402 (LG 8)	*206*	*206*	*206*	*206*	*206*	*206*	*206*	*206*
Z9637 (LG 8)	*146*	*146*	*146*	*146*	*146*	*146*	*146*	*146*
Z106 (LG 9)	*188*	*188*	*188*	*188*	*188*	*188*	*188*	*188*
Z1450 (LG 10)	*238*	*238*	*258*	*238*	*238*	*238*	*238*	*238*
Z10215 (LG 11)	*118*	*118*	*118*	*118*	*118*	*118*	*118*	*118*
Z7576 (LG 12)	*121*	*378*	*376*	*121*	*378*	*378*	*206*	*206*
Z6622 (LG 13)	*154*	*154*	*154*	*274*	*154*	*274*	*274*	*274*
Z1652 (LG 14)	*140*	*98*	*98*	*98*	*140*	*140*	*98*	*140*
Z3309 (LG 15)	*104*	*118*	*174*	*176*	*118*	*118*	*176*	*118*
Z992 (LG 16)	*220*	*256*	*256*	*256*	*220*	*256*	*220*	*256*
Z3127 (LG17)	*138*	*140*	*138*	*140*	*140*	*140*	*140*	*140*
Z1990 (LG17)	*190*	*190*	*190*	*190*	*190*	*190*	*190*	*190*
Z1136 (LG 18)	*116*	*116*	*116*	*116*	*184*	*116*	*116*	*116*
Z9331 (LG 19)	*292*	*292*	*292*	*292*	*292*	*292*	*292*	*292*
Z9708 (LG 20)	*218*	*218*	*218*	*218*	*218*	*218*	*218*	*218*
Z11113 (LG 21)	*220*	*220*	*220*	*220*	*196*	*220*	*220*	*220*
Z6613 (LG 22)	*184*	*118*	*184*	*184*	*184*	*184*	*184*	*184*
Z5265 (LG 23)	*196*	*216*	*216*	*200*	*152*	*216*	*196*	*216*
Z7349 (LG 24)	*204*	*212*	*212*	*212*	*212*	*212*	*212*	*212*
Z11786 (LG 25)	*164*	*164*	*164*	*152*	*152*	*164*	*164*	*164*

Abbreviation: LG, linkage group.
[a] See Shimoda *et al.* [15].

Table S5

Summary of amplified fragment length polymorphism (AFLP) data.

	No. of fish	No. of primer sets	No. of bands	Frequency of polymorphisms (%)	BSI[a]
Clonal line	8	64	34–94	0.00	1
Intact control	8	31	52–103	50.00–92.42	0.76 ± 0.13

Abbreviation: BSI: band sharing index

[a] The data are shown as total means ± SDs for all primers. See Arai *et al.*[32].

Table S6

Adaptors and primers used for amplified fragment length polymorphism (AFLP).

Adaptor or primer	Sequences (5′→ 3′)
*Eco*RI adaptor-1	CTCGTAGACTGCGTACC
*Eco*RI adaptor-2	AATTGGTACGCAGTCTAC
*Mse*I adaptor-1	GACGATGAGTCCTGAG
*Mse*I adaptor-2	TACTCAGGACTCAT
*Eco*RI preselective primer	GACTGCGTACCAATTCA
*Mse*I preselective primer	GATGAGTCCTGAGTAAC
Selective primer:	
E-AAC	GACTGCGTACCAATTCAAC
E-AAG	GACTGCGTACCAATTCAAG
E-ACA	GACTGCGTACCAATTCACA
E-ACC	GACTGCGTACCAATTCACC
E-ACG	GACTGCGTACCAATTCACG
E-ACT	GACTGCGTACCAATTCACT
E-AGC	GACTGCGTACCAATTCAGC
E-AGG	GACTGCGTACCAATTCAGG
M-CAA	GATGAGTCCTGAGTAACAA
M-CAC	GATGAGTCCTGAGTAACAC
M-CAG	GATGAGTCCTGAGTAACAG
M-CAT	GATGAGTCCTGAGTAACAT
M-CTA	GATGAGTCCTGAGTAACTA
M-CTC	GATGAGTCCTGAGTAACTC
M-CTG	GATGAGTCCTGAGTAACTG
M-CTT	GATGAGTCCTGAGTAACTT

Table S7

Primer combinations for selective amplification using amplified fragment length polymorphism (AFLP)[a].

	M1<br>M-CAA	M2<br>M-CAC	M3<br>M-CAG	M4<br>M-CAT	M5<br>M-CTA	M6<br>M-CTC	M7<br>M-CTG	M8<br>M-CTT
E1<br>E-AAC	E1/M1*	E1/M2*	E1/M3*	E1/M4*	E1/M5*	E1/M6*	E1/M7*	E1/M8*
E2<br>E-AAG	E2/M1*	E2/M2*	E2/M3*	E2/M4*	E2/M5*	E2/M6*	E2/M7*	E2/M8*
E3<br>E-ACA	E3/M1	E3/M2	E3/M3	E3/M4	E3/M5	E3/M6	E3/M7	E3/M8
E4<br>E-ACC	E4/M1	E4/M2	E4/M3	E4/M4	E4/M5	E4/M6	E4/M7	E4/M8
E5<br>E-ACG	E5/M1	E5/M2	E5/M3	E5/M4	E5/M5	E5/M6	E5/M7	E5/M8
E6<br>E-ACT	E6/M1	E6/M2	E6/M3	E6/M4	E6/M5	E6/M6	E6/M7	E6/M8
E7<br>E-AGC	E7/M1	E7/M2*	E7/M3*	E7/M4*	E7/M5*	E7/M6*	E7/M7*	E7/M8*
E8<br>E-AGG	E8/M1*	E8/M2*	E8/M3*	E8/M4*	E8/M5*	E8/M6*	E8/M7*	E8/M8*

[a] For the clonal line, all of the combinations were used; for the intact control, the combinations with asterisk in the table were used.
